# Effect of Hydroxyl-Containing Fragments on the Structure and Properties of Membrane-Forming Polyamide-Imides

**DOI:** 10.3390/membranes13080716

**Published:** 2023-08-01

**Authors:** Svetlana V. Kononova, Galina K. Lebedeva, Galina N. Gubanova, Elena V. Kruchinina, Elena N. Vlasova, Nadezhda V. Afanas’eva, Elena N. Popova, Anatoly Ya. Volkov, Elena N. Bykova, Natalya V. Zakharova

**Affiliations:** Institute of Macromolecular Compounds, Russian Academy of Science, Bolshoy pr. 31, Saint-Petersburg 199004, Russia; constanta2011.lebedeva@mail.ru (G.K.L.); gubanovagn@yandex.ru (G.N.G.); evkruchinina@mail.ru (E.V.K.); evl021960@gmail.com (E.N.V.); afann@mail.ru (N.V.A.); popovaen@hq.macro.ru (E.N.P.); volanya@bk.ru (A.Y.V.); bykova.elena.n@gmail.com (E.N.B.); na_zar@inbox.ru (N.V.Z.)

**Keywords:** polyamide-imides, hydrophilic polymers, polycondensation, structure, thermogravimetric analysis, dielectric spectroscopy, pervaporation

## Abstract

The structural features and thermophysical and transport properties of dense nonporous membranes of the casting type from (co)polyamide-imides synthesized by the polycondensation of the diacid chloride of 2-(4-carboxyphenyl)-1,3-dioxoisoindoline-5-carboxylic acid and diamines 5,5′-methylene-bis (2-aminophenol) (DADHyDPhM) and 4,4′-methylenebis(benzeneamine) (DADPhM), taken in molar ratios of 7:3, 1:1, and 3:7, have been studied. The effect of hydroxyl-containing modifying fragments of dihydroxy diphenylmethane introduced in various amounts into the main polymer chain on the pervaporation properties of the formed films is discussed. It has been shown that the presence of the residual solvent N-methyl-2-pyrrolidone in the films not only has a plasticizing effect on the characteristics of film membranes but also promotes the preferential transmembrane transport of polar liquids, primarily methanol (permeation rate over 2 kg for a copolymer with a ratio of DADHyDPhM:DADPhM = 7:3). The removal of the residual solvent from the polymer film, both thermally (heating to 200 °C) and by displacement with another solvent as a result of sequential pervaporation, led to a significant decrease in the rate of transfer of polar liquids and a decrease in the selectivity of the membrane. However, the dehydrocyclization reaction resulted in more brittle films with low permeability to penetrants of different polarities. The results of our comprehensive study made it possible to assume the decisive influence of structural changes in membranes occurring in connection with the competitive formation of intra- and intermolecular hydrogen bonds.

## 1. Introduction

The development of new polymeric membranes of a complex morphology remains an urgent task, despite the fact that a number of polymers of various classes are already being used in membrane technologies. Many target-purpose membranes are designed as multicomponent systems, which makes it possible to avoid the shortcomings of individual polymers and achieve the desired membrane properties by including other components [1,2]. However, the synthesis of polymeric materials containing macromolecular fragments that differ significantly in chemical structure is one of the most promising ways to design membrane materials. In this case, a single polymer can be used to form a material that combines the properties of pristine polymers. It is possible to synthesize copolymers containing fragments of homopolymers with completely different functional properties and characteristics. For example, together with fragments of high rigidity, flexible fragments can be introduced into the composition of a macromolecule. A copolymer may include blocks with reactive groups of atoms in conjunction with blocks that do not exhibit functional activity; another example is a macromolecule with well-solvated fragments and the parts that are not solvated in the same solvents (hydrophilic and hydrophobic, etc.). This approach to polymer design can yield non-porous polymeric membranes of complex morphology, which can be characterized by both micro- and macro-heterogeneous supramolecular structures. This gave significant positive results in the development of proton-conducting, pervaporation, and gas separation membranes [3,4,5,6,7,8,9,10,11,12,13].

In the first case, the amphiphilic polymers with hydrophilic blocks containing strongly dissociating polar groups were used to produce proton-conducting membranes with heterophase structures. These membranes included transport channels in which ion-exchange centers were concentrated [14]. This structure promoted the preferential transmembrane transport of hydrogen or hydronium ions [15]. Also of interest are heterophase systems that effectively provide the predominant transport of gases or vapors capable of strong adsorption interactions with the membrane-forming polymer (the second case) [15]. To achieve the maximum implementation of the separating properties of these membranes, it is important to form a micro heterogeneous structure that contains an extended amorphous microphase highly permeable for one of the components of a separated mixture. This microphase should be surrounded with another phase of densely packed polymer chains. The second structural feature is also required since the formation of this dense polymeric region, in which there is a greater resistance to the flow of separated substances, prevents swelling in the components of the mixture to be separated in another permeable polymeric region of the membrane. Multiblock poly(ether-urea-imide)s that are highly selective in the separation of gas mixtures are good examples of such systems [7]. In this paper, multiblock copolymers containing polyethylene oxide (PEO) fragments are considered good candidates for membrane separation of CO_2_-containing mixtures. The polyethylene oxide microphase possesses a strong affinity for CO_2_, while the presence of the polyimide phase with low permeability improves the mechanical properties of the membrane. However, when forming film membranes from this material, the authors encountered another problem, namely, the crystallization of PEO and the weak mechanical properties of the block copolymer at a high PEO concentration. The authors suggested some ideas for understanding and optimizing the behavior of multiblock copolymers with high PEO contents as highly selective membranes for CO_2_/N_2_ separation and proposed a way to avoid crystallization of PEO blocks.

The copolymers consisted of soft polyesteramine blocks and very hard urea imide blocks to provide improved physical crosslinking and excellent film-forming ability. CO_2_ permeability increased significantly with increasing soft block content due to strong phase separation. The block copolymer with the highest soft block content was a thermoplastic elastomer in which soft blocks formed well-separated highly permeable percolating nanodomains. The copolymer with the lowest soft block content was a hard glassy material with very little phase separation. The important point is that the increase in CO_2_ gas diffusion was much higher than the increase in CO_2_ sorption with a rise in soft block content. The separating properties of multiblock copolymers of the polyurethane imide class are mainly controlled by gas diffusion. 

Multiblock (segment) polyetherimide was obtained by the polycondensation of pyromellitic dianhydride (PMDA) and p-phenylenediamine in the presence of a mixture (taken in a molar ratio of 10:1) of polybutylene adipate (Mn = 1 × 10^3^) terminated with hydroxyl groups and polyethylene adipate (Mn = 1 × 10^4^) terminated with hydroxyl groups. The copolymer synthesized in [8] formed strong elastic films with high values of ultimate strain at failure, although films of poly(N,N’-phenylene)pyromellitimide (a related polymer whose derivative is the synthesized multiblock polyester-imide) are characterized by extreme brittleness due to their high crystallinity (εp < 1%) [16]. The synthesized block copolymer resembled thermoplastic elastomers in terms of its viscoelastic properties [8]. Non-porous film membranes were tested while separating benzene or toluene from their mixtures with n-octane or cyclohexane under pervaporation conditions. When the feed mixture containing equal amounts of aromatic and aliphatic hydrocarbons (50 wt.%) was separated using the polyester-imide membrane, the resulting permeate contained 92 wt.% toluene and 8 wt.% n-octane and 90 wt.% benzene and 10 wt.% cyclohexane, respectively. The separation factor for the toluene/n-octane mixture was 10 at a permeability of 2.3 kg·µm·h^−1^·m^−2^, while the benzene/cyclohexane mixture’s separation factor was 7 at a permeability of 5.2 kg·µm·h^−1^·m^−2^.

Taking into account the choice of polyester and imide blocks in multiblock polyester-imides [17,18,19], the transport of liquid substances in the pervaporation separation of aromatic/aliphatic hydrocarbon mixtures occurs in polyester microdomains. The swelling of these microdomains is limited by the hard phase (imide segments). At the same time, the length of the polyester blocks can affect the characteristics of membranes; e.g., the membranes based on polymers with shorter polyester blocks have lower specific flux values but higher separation factors.

The selectivity and permeability ratios of multiblock copolymers containing flexible blocks of different lengths in the main chains have been investigated [20,21]. Although the extent to which the transport properties of block copolymer membranes are dependent on their structure has been studied at various sites [7,22,23], no systematic research work has been carried out. The introduction of hydrophilic groups into a hydrophobic macromolecule is an important technique for the formation of highly selective diffusion membranes. The most pronounced effects associated with the formation of a microheterogeneous polymer structure in which hydrophilic and hydrophobic nanosized phases can be distinguished were observed in membranes based on tetrofluoroethylene copolymers containing sulfated block fragments [14,15,24,25,26]. The membranes consisting of fluorine-containing block polyacids were used in the separation of liquids [27,28,29,30,31]. In particular, the efficiency of evaporation during the dehydration of sulfuric acid with Nafion-112^®^ and Nafion-117^®^ membranes was evaluated both at 100 and 120 °C. In all experiments, the membranes were found to give significant water fluxes with high selectivity [28].

Since Nafion has accessible sulfonic groups in its structure, the authors of [32] proposed the idea of modifying it with primary amines, and they did so using aniline, benzylamine, and ethanolamine. The pervaporation experiments involving mixtures of methanol and dimethyl carbonate showed that Nafion modified with ethanolamine is about twice as selective for methanol as pure Nafion, Nafion sodium salt, and Nafion modified with benzylamine. All amine-modified Nafions showed a lower sorption of water vapor, methanol, and dimethyl carbonate than those composed of pure Nafion and its sodium salt [32].

The data on pervaporation in the ethanol–water–Nafion system showed that water predominantly penetrates through the membrane, despite the preferential sorption of alcohols. The significant role of the counterion of the sulfo group in the transport properties of the membrane has been established. The interaction between the permeant and the polymer in the Nafion membrane with Na^+^ counterions is two times stronger than that in the polymer containing (CH_3_)_3_NH^+^ as a counterion [29,30,31]. Block copolymers of tetrafluoroethylene with monomers containing sulfonic acid groups as well as copolymers modified by sulfonic acid groups (which retain the block structure after the reaction in chains or other modifications by sulfo groups) were tested in pervaporation processes. They demonstrated the effective transfer of polar components of separated mixtures [33]. Nafion was modified by exposing it to an excess of (a) simple inorganic bases such as ammonium and hydroxide; (b) simple organic bases such as tris(hydroxymethyl)aminomethane, ethanolamine, and benzylamine; and (c) amino acid derivatives. Dynamic mechanical analysis (DMA) measurements showed shifts in the β-transition temperatures of these modified polymers. Therefore, the β-transition was found to be mainly associated with the side chains and not with the main chain of polytetrafluoroethylene (PTFE). The temperature of the α-transition, that is, the glass transition, in some modifications shifted significantly (from about 97 °C for Nafion to about 210 °C for its derivatives). The authors suggested that this change was caused by charge delocalization in the substituted sulfo groups; a similar trend (sharp increase) was observed for the decomposition temperature.

Similar observations were made in our previous studies concerning polycondensation block copolymers with sulfonic acid groups in one of the blocks and their triethylammonium salts [34]. The structural and morphological features of sulfonated copolyimides with limited chain mobility and their influence on the transport properties of the material were discussed. The glass transition temperature of the copolymer increases when the size and charge density of the counterion decrease and increase, respectively. According to the results of morphological studies, the synthesized block copolymer contains interconnected polar phase regions within a non-polar matrix. The influence of NH^+^(Et)_3_ ions on the copolymer solubility was established. The morphological features of the block copolymer enable the selective facilitated transport of penetrants in the pervaporation separation of various binary methanol mixtures. The polymer showed good separating properties in the pervaporation separation of near-azeotropic MeOH/DMC and MeOH/DME mixtures [34].

In [11], we reported a study on copolymers with weakly dissociating acid groups using copolyimide-imides containing hydrophilic fragments of diaminobenzoic acid as an example. In addition to the units containing fragments of diphenyl oxide with a flexible -O- hinge, rigid links with carboxyl groups of diaminobenzoic acid (without hinges) were introduced into the macromolecule. The chemical structure of the copolymers and the structure of the corresponding film membranes were studied. On the basis of experimental data and our knowledge of the close reactivities of the monomers entering into the co-polycondensation reaction, a conclusion was made about the statistical distribution of monomer units in the polymer chain. In this case, the molar ratio of monomers used in the synthesis determined the presence of extended blocks in the macromolecule. In other words, when comonomeric diamines of diaminodiphenyl ether and diaminobenzoic acid were loaded in a 3:7 ratio, long blocks containing carboxyl groups appeared in the synthesized polymer, and when the monomer ratio was equal to 7:3, extended hydrophobic blocks were present in the copolymer.

In addition, the AFM studies in the phase contrast mode revealed two clearly visible phases: the polymer phase and the solvent phase covering each domain. According to thermogravimetric analysis, the amount of residual solvent reached 10 wt.%. The transport properties of the synthesized polymers correlated well with the obtained characteristics of the membrane surface, their domain structure, and the presence of strongly bound residual solvent. The results clearly demonstrated that all tested membranes were stable under pervaporation conditions. The transport of cyclohexane through these films remained virtually unchanged after a cycle of pervaporation experiments involving polar liquids.

The fluxes of polar penetrants through a homopolymer containing no carboxyl groups and a copolymer containing 30% diaminobenzoic acid fragments were practically similar when penetrants were successively passed through membranes in the following series: cyclohexane → ethanol → methanol → water. At first glance, this seems strange, since the introduction of more rigid fragments into the macromolecule is accompanied by an increase in Tg. This effect usually leads to a decrease in the diffusion permeability of nonporous membranes due to a decrease in the mobility of molecular chains. However, in the case of the copolymers studied in this work, there were additional factors affecting the transport properties of the membranes. This effect has a logical explanation, since the copolymer included extended blocks that did not contain carboxyl groups. However, it remains unclear why, in the second part of the cycle, after passing water through the membrane, the copolymer was “under the influence” of carboxyl-containing fragments. At the same time, in the second part of the cycle (water → methanol → ethanol → cyclohexane), the flow of methanol through the carboxyl-containing homopolymer decreased while the flow of methanol through the copolymer increased. This effect is possibly associated with the domain structure, which is present in the investigated polymers.

Another attempt to control the hydrophilic–lipophilic balance of a polymer membrane was made in one of our more recent works [13] by introducing hydroxyl-containing fragments into the polyamide-imide macrochain. In this work, the novel modification of polyamide-imides was carried out; the fragments (units) containing hydroxyl (hydrophilic) groups in the ortho-position to the amide bond were introduced into the macromolecule. New polymers were synthesized (polyamide-imides and poly(amide-o-hydroxy-imides, as well as copolymers of the amide-o-hydroxy-imides class), and their properties (structure, transport properties during the pervaporation of liquids of different polarities, etc.) were comprehensively studied. To obtain target polymers, several structurally similar diamines that differed only in the amounts of hydroxyl groups (and diamines without hydroxyl groups) were purposefully chosen as monomers. As a result, it was possible to vary the number of hydroxyl functional groups in the polymer chain. Due to the presence of hydroxyl groups in the ortho-position to the amino groups (which participate in polycondensation), carrying out further transformations in the polymer chain is a possibility, i.e., cyclization with the formation of hydrophobic fragments in the polymer. Our intention was to use these reactions as a means of controlling not only the hydrophilicity of the resulting polymers but also their useful properties, including their mechanical and transport characteristics. It was shown that the amount of hydroxyl groups can be controlled by the reaction conditions.

The behavior of dense films of polyamide-imides during the pervaporation of liquids has been studied. The choice of the temperature regime for the preparation of films was based on information about the change in the properties of these polymer films upon heating (homopolymer samples heated to 150 °C exhibited plastic deformation). Nonporous films prepared in this way turned out to be suitable materials for the formation of diffusion membranes. It should be noted that a small amount of a film-forming solvent, which was detected by various methods, contributed to the properties of the membrane films. This effect was observed in an earlier work of ours that was devoted to the formation of diffusion membranes from aromatic polyamide-imides [35]. Presumably, the stability and properties of membranes of this type are associated with the presence of a residual solvent (N-methyl-2-pyrrolidone) physically bound to the polymer. It was shown that this solvent was completely removed only when heated at temperatures above 200 °C.

The pervaporation fluxes of various substances (penetrants) through membranes obtained by annealing at 100 or 150 °C were measured. We studied the pervaporation transport of the following individual substances of different polarities at 40 °C: cyclohexane, ethanol, methanol, and water. The experiments showed that the polyamide-imide films prepared by casting solutions onto a substrate and removing the solvent via evaporation at 100 °C did not have stable transport characteristics. Annealing the films at 150 °C led to the stabilization of membrane properties; this effect is associated with the removal of significant amounts of the residual solvent. Heating above 150 °C did not cause any changes in the chemical structure of the polymers. As expected, an increase in the number of hydroxyl groups in the polymer generally led to an increase in the water permeability of the membranes. The permeability of cyclohexane remained tendentiously low. No dependence of the permeability of alcohols on the amount of hydroxyl groups in the copolyimides was found for any of the studied polymers.

An analysis of the above-listed results suggested that the films based on the synthesized polymers and the structural changes caused by the removal of the solvent and dehydrocyclization were worthy of further investigation. The work raised many questions. It remains unclear whether the supposed competing processes involving hydroxyl groups (formation and breaking of hydrogen bonds and dehydrocyclization) occur. The issue of the influence of these processes on the mechanical and thermophysical characteristics of the studied polymers, as well as their film-forming properties, requires further consideration. The most interesting question is that of the supramolecular structure of membranes since the synthesized copolymers contain long fragments that differ significantly in the number of hydroxyl functional groups. This brings up another question: how does the formed structure affect the polymer film’s ability to perform the mass transfer of liquids (penetrants) of different polarities? First of all, this concerns the liquids capable of physical interaction with the hydroxyl groups of polymers and the formation of hydrogen bonds. For the present study, we examined the influence of the synthesized polymers’ chemical structure on the morphology and properties of the film membranes. 

## 2. Materials and Methods

### 2.1. Preparation of Samples for the Studies

#### 2.1.1. Reagents

4,4′-Methylenebis(benzeneamine) (4,4′-Diaminodiphenylmethane, DADPhM) and Propylene oxide (99%) were of reagent grade, purchased from Sigma-Aldrich (St. Louis, MO, USA), and used without additional purification. N-Methyl-2-pyrrolidone (N-MP, St. Louis, MO, USA) was used without further purification.

5,5′-Methylenebis(2-aminophenol) (4,4′-Diaminodihydroxydiphenylmethane, DADHyDPhM) was prepared as described in [36]. DADPhM and DADHyDPhM were dried for 48 h, and subsequently, the reagents were dried under vacuum.

Diacid chloride of (2-(4-Carboxyphenyl)-1,3-dioxoisoindoline-5-carboxylic acid) (diacid chloride of CPDCA) was prepared according to the technique described in [37]. Before polycondensation, the diacid chloride of CPDCA was dried for 24 h. 

#### 2.1.2. Polymer Preparation

**Figure 1:**
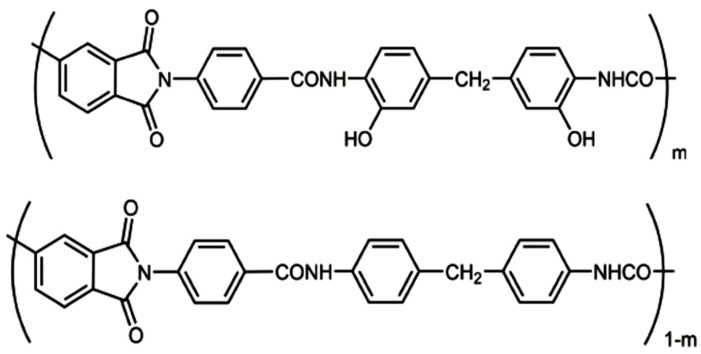
Chemical structures of Hy-PAI I (m = 1), Hy-coPAI’s II–IV (m = 0.7, 0.5, 0.3), and PAI V (m = 0).

Poly(o-hydroxyamide-imides) (Hy-PAI I and Hy-coPAI’s II–IV) and Poly(amide-imide) (PAI V) with the chemical structures shown in Figure 1 were synthesized according to a previously described procedure [13,38]. 

All of the polymers in Figure 1 were prepared in a similar way, via low-temperature polycondensation in a solution (N-MP as solvent) of the diacid chloride of CPDCA and DADHyDPhM (Hy-PAI I) or DADPhM (PAI V), or a mixture of diamines DADHyDPhM and DADPhM with various molar ratios between components (7:3 for Hy-coPAI II, 1:1 for Hy-coPAI III, and 3:7 for Hy-coPAI IV).

Powder-like polymer samples were isolated from the initial solution via precipitation into water. The obtained polymer was precipitated, and afterwards, the liquid was decanted and the precipitate was washed with a small amount of ethanol. The reaction product was filtered through a Schott filter. The polymer was dried at 70 °C in an oven and then at 50 °C in a vacuum dryer to a constant weight.

#### 2.1.3. Membrane Formation

Dense PAI’s (I–V) polymer films were prepared by casting 15 wt.% polymer solutions in N-MP onto a glass plate followed by stepwise drying at 50–100 °C in order to remove the solvent. The obtained films were separated from the glass plate, subjected to thermal treatment at 150 °C, and then heated stepwise up to 200 °C or 300 °C (with each step being equal to 50 °C).

### 2.2. Instruments and Methods

#### 2.2.1. Determination of the Molecular Weight and Hydrodynamic Characteristics of Polymers

**Figure 2:**
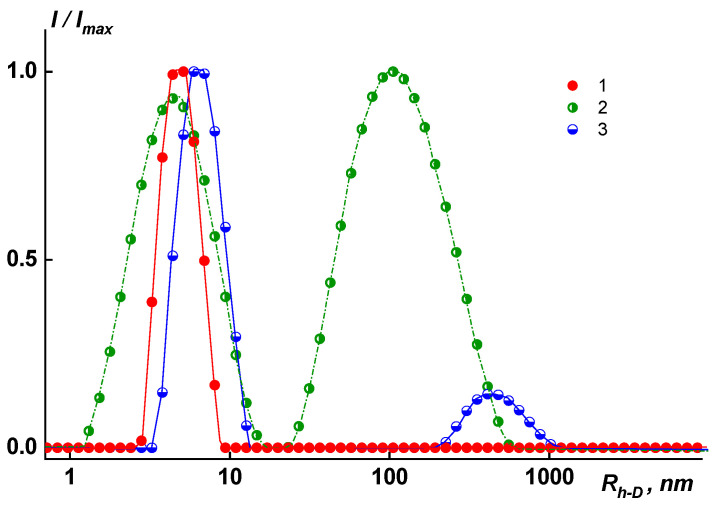
Relative intensity *I/I_ma_*_x_ of scattered light vs. the size of the scattering species *R*_h-D_ for the copolymers (T = 21 °C in N-MP solutions): 1. Hy-coPAI IV, *c* = 0.4780 g/cm^3^ or 0.0048 g/dL; 2. Hy-coPAI III, *c* = 0.8259 g/dL or 0.008381 g/cm^3^; 3. Hy-coPAI II, *c* = 0.4423 g/dL or 0.0044 g/cm^3^. *I_ma_*_x_ is the maximum light scattering intensity value for a given solution.

**Figure 3:**
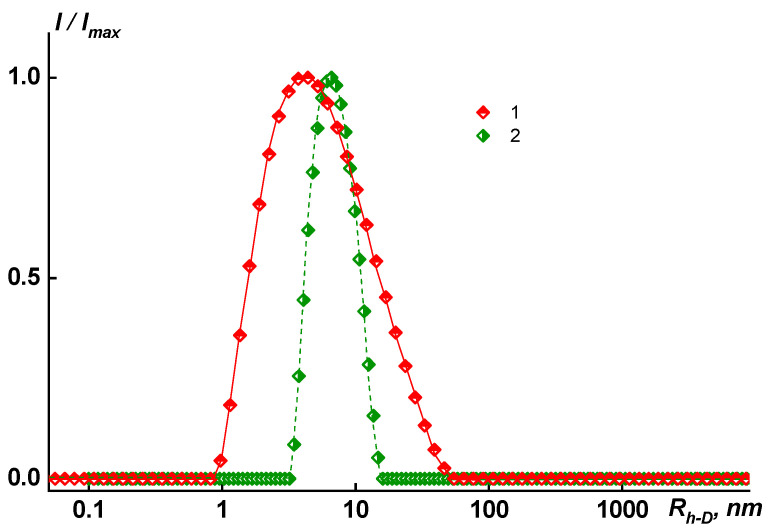
Relative intensity *I/I_ma_*_x_ of scattered light vs. the size of scattering species *R*_h-D_ in N-MP and T = 21 °C for 1. PAI I, *c* = 2.4016 g/dL or 0.0240 g/cm^3^; 2. PAI V at *c* = 1.5756 g/dL or 0.0158 g/cm^3^. *I_ma_*_x_ is the maximum light scattering intensity value for a given solution.

The molecular masses (MM) and hydrodynamic radii (*R*_h–D_) of the (co)polymers were measured using static and dynamic light scattering methods in dilute solutions in N-methyl-2-pyrrolidone and in mixed solvents at 21.0 °C. Light scattering was conducted on a Photocor Complex unit (Fotokor LLC, Moscow, Russia); a Photocor-DL diode laser served as the light source (power of 5–30 mW, wavelength λ = 659.1 nm). The unit was calibrated by using benzene (RV = 1.38 × 10^−5^ cm^−1^). The correlation function of the scattered light intensity was obtained using a Photocor-PC2 correlator with 288 channels and was processed with DynalS software (ver. 8.2.3, SoftScientific, Tirat Carmel, Israel). In these solutions, the asymmetry of light scattering was absent; thus, the *M*_w_ of the copolymers was determined using the Debay method. The data were processed using the cumulant method and the Tikhonov regularization procedure. The hydrodynamic radii *R*_h-D_(*c*) of the scattering particles at concentration *c* were calculated using the Stokes–Einstein equation:

*R*_h-D_(*c*) = k*T*_a_/6π*η*_0_*D*_0_ (1)

where *T*_a_ is the absolute temperature, *D*_0_ is the diffusion coefficient of particles, and k is the Boltzmann constant. The dynamic light scattering studies detected one mode for the investigated polymer solutions PAI V, PAI I, and Hy-coPAI’s IV (m = 0.3) in the N-MP or mixed solvents (Figure 1). The fraction of aggregates (*R*_h-s_ = 114 _HM_) in the solutions of Hy-coPAI III (m = 0.5) sample was only 0.24 wt.% and 0.02 wt.% for the copolymer Hy-coPAI’s II (m = 0.7) with *R*_h-s_ = 446 nm. Therefore, their Mw was determined based on the contribution of individual macromolecules—*R*_h-f_ = 5.6 nm and 6.5 nm, respectively, to the overall intensity of the polymer solution (Figure 2 and Figure 3, Table 1). The hydrodynamic radii *R*_h-D_ of the macromolecules were obtained as the average values of *R*_h-D_(*c*). The hydrodynamic radius of the particles at room temperature practically does not depend on the concentration (within the error) for all of the copolymers in the N-MP and mixed solvents. The refractive index increments dn/dc were measured using a Refractometer RA-620 (Kyoto Electronics Manufacturing, Tokyo, Japan). The thermodynamic quality of the solvents used for these systems is good.

#### 2.2.2. IR Spectroscopy Study

The IR spectra of the polymer films were recorded on a Bruker Veryex 70 IR Fourier spectrometer at room temperature in the range of 400–4000 cm^−1^ using a Pike single attenuated total internal reflection (ATR) microattachment with a working element made of ZnSe. When registering the ATR spectra, a correction was introduced that takes into account the penetration depth depending on the wavelength.

Powders of the individual polymers Hy-PAI I and PAI V and the Hy-coPAI III copolymer with a component ratio of 1:1 were studied via IR spectroscopy and warmed up to 300 °C. The spectra were recorded with a resolution of 4 cm^−1^; the number of scans was 30.

#### 2.2.3. X-ray Diffraction (XRD) Analysis

All XRD experiments were performed using a Seifert XRD 30003TT X-ray diffractometer equipped with a primary monochromator. We used CuKα radiation with λ = 1.54056 Å, and the scans were conducted at room temperature. Film diffraction pictures were taken at a 2θ angle range from 5 to 40° with a step interval of 0.050° and a count time of 10 s/step.

#### 2.2.4. Thermal Analysis

In order to establish the thermophysical characteristics of coPAI’s and PAI I, V homopolymers, TGA, DSC, and DMA were used to study the samples heat-treated at 150 °C (a series of pervaporation tests for such membrane samples were carried out) and 300 °C, for which there was a partial dehydrocyclization.

*Differential scanning calorimetry (DSC)* analysis of the samples was carried out using a Netzsch DSC 204 F1 (Erich NETZSCH GmbH & Co. Holding KG, Selb, Germany) heat flow differential scanning calorimeter in a temperature range ranging from 0 to 400 °C in an argon atmosphere (argon flow rate: 25 mL·min^−1^; heating rate: 10 deg·min^−1^). 

*Thermal-gravimetric analysis* was performed using the TG 209 F1 Libra® Thermogravimetric Analyzer (Erich NETZSCH GmbH & Co. Holding KG, Selb, Germany)) setup in a temperature range ranging from 30 to 600 °C at the same heating rate in an argon medium. The weight of the tested samples was 2–3 mg.

#### 2.2.5. Density Determination by Flotation Method

The density of the studied materials at a temperature of T = 20 °C was determined by using the flotation method on small fragments of the samples in mixtures of carbon tetrachloride and toluene.

#### 2.2.6. Solution Density, Specific Partial Volumes, and Buoyancy Factors 

The buoyancy factors of the samples were calculated from the densities of the copolymer solutions, which were measured using a Kyoto Electronics DA-640 densitometer (Kyoto Electronics Manufacturing, Tokyo, Japan). The respective specific partial volumes (and buoyancy factors (1-υρ_0_), where ρ—the density) were determined for the homopolymers and three samples of the copolymers presented in Table 2. The measurements were performed in N-MP in a concentration range of 0.0013–0.0088 g/cm^3^ (the error was no more than 10%). Buoyancy factor (1-υρ_0_) was calculated from the slope of the dependence of the density of (co)polymer solutions on their concentration. 

Density measurement results—specific partial volume and buoyancy factor—are presented in Table 2.

#### 2.2.7. Dielectric Spectroscopy Method

Measurements of the dielectric loss tangent tgδ and capacitance were carried out using an Automatic LCR Meter WAYNE KERR 4270. The error was 2%.

To study temperature–frequency dependence, the sample, which is a disk 10–20 mm in diameter and 15–100 µm thick, was fixed in a two-electrode holder, which was placed in a glass measuring flask with platinum inlets. The flask was evacuated and then filled with dry air. The dry air was obtained by passing through series-connected vessels with sulfuric acid and phosphorus pentoxide. The measurements were carried out in a temperature range ranging from −60 °C to 160 °C and at frequencies ranging from 120 to 910,000 Hz.

Dielectric spectroscopy is one of the most effective methods for studying the processes associated with molecular mobility in a polymer sample placed in an external alternating electric field. It can be used to obtain data on the molecular mobility of a macromolecule as a whole or segments of the main chains, bulky side substituents, etc.

Studying dielectric polarization involves determining the equilibrium and non-equilibrium (transitional) parameters of the medium. Equilibrium parameters (for example, static permittivity) are directly related to characteristics such as dipole moment and polarizability, which are determined by short-range interactions in a molecular medium. 

#### 2.2.8. Pervaporation Experiments

The pervaporation properties of the obtained dense membranes (nonporous films) were tested for different penetrants (water, ethanol, methanol, cyclohexane) using a non-continuous flow laboratory cell, as described in [11,34], with an operating membrane area of 1.38 × 10^−3^ m^2^ at a constant temperature of 40 °C. Permeate vapors were condensed using liquid nitrogen. The received permeate was weighed, and the flux value *J* [kg⋅m^−2^⋅h^−1^] was estimated using the following equation:

(2) $$J=m\times S^{-2}\times t^{-1}$$

where *m* is the mass [kg] of the penetrant permeated through membrane area *S* [m^2^] in a period *t* [h]; P = *J* · *l* [μm⋅kg⋅m^−2^⋅h^−1^] (permeation rate) is the flux of a penetrant normalized to a membrane thickness of 1 μm.

During the experiment, we studied the membrane by sequentially passing liquids (penetrants) of different polarities through it and drawing comparisons using chromatographic methods as described by in our previous works, for example, in [11]. The fluxes of the penetrants were measured in the following sequence: at the beginning of the experiment in the sequence (I) cyclohexane, ethanol, methanol, water—as the polarity increased (symbolically indicated by a → arrow); further in the sequence (II) water, methanol, ethanol, cyclohexane—as the polarity decreased (conventionally indicated by a ← arrow). At each stage, after studying the membrane permeability of a certain solvent, the membrane was dried, controlling the test on a dry membrane for the stability of the residual pressure in the chamber under the membrane in the pervaporation cell. This test showed that the membrane does not contain a solvent not bound to the polymer, the vapors of which could be released during pervaporation. Such a conditionally cyclic technique made it possible to obtain information about the state of the polymeric material of the membrane after each stage of pervaporation. For example, if, at the beginning of the experiment (sequence (I)), the flux of cyclohexane was approximately equal to the flux of this solvent at the end of the cyclic experiment (sequence (II)), the membrane material practically did not undergo any irreversible changes. This technique is still being finalized and improved by us as we work with various materials; however, if one wishes to predict the behavior of these materials (in particular, polymers) in real pervaporation processes, in its present form, it can be very informative when working with new materials.

The composition of the initial mixtures (feed) and permeates was analyzed using gas-liquid chromatography (GS-2014 Shimadzu gas chromatograph equipped with a Porapak™ Q packed GC column (SUPELCO), 3 m in length, 2.0 mm inner diameter) and refractometry.

## 3. Results and Discussion

In this work, we studied the structure and properties of (co)polymers synthesized according to a procedure previously developed by us in a prior work [13]; we also studied the products of their modification via thermal dehydrocyclization. A specific feature of this series of (co)polymers is the decrease in the number of hydroxyl groups in macromolecules upon passing from Hy-coPAI I (m = 1) to Hy-coPAI II–IV (m = 0.7, 0.5, 0.3) and PAI V (m = 0). At the same time, PAI V differs from the other polymers in this series due to its complete absence of hydroxyl groups.

It should be noted that the synthesis of all polymers was carried out under the same conditions of the low-temperature polycondensation reaction. The reactivity of the diamines introduced into the reaction is somewhat different since the presence of hydroxyl groups in the o-position to the amine groups leads to a shift in the electron density of the monomer and weakens its nucleophilic ability.

**Figure 4:**
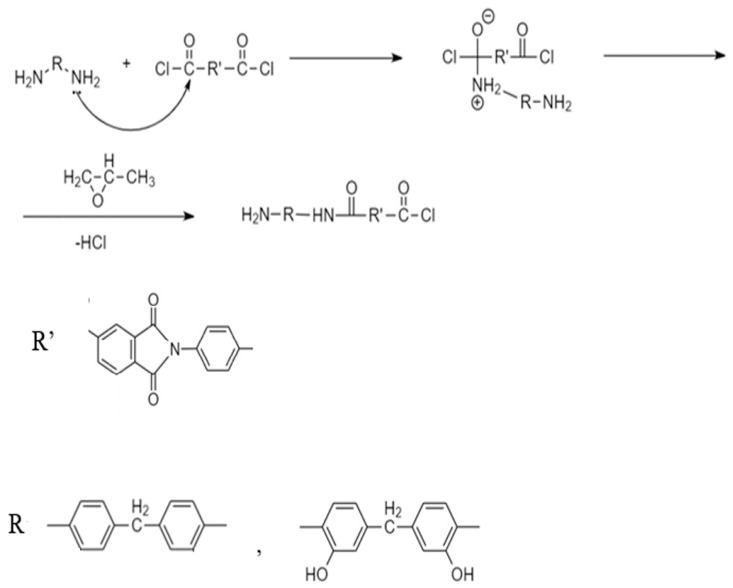
Polycondensation reaction mechanism for producing (co)polymers.

At the same time, introduction into the reaction zone diamines (shown in Figure 4), in equal amounts (Hy-coPAI III, m = 0.5), should result in a copolymer containing short blocks corresponding to PAI V. At the same time, the stepwise nature of the polycondensation reaction contributes to the production of a random copolymer. Determining the actual distribution of hydroxyl-containing fragments in the polymer chain is, in this case, an extremely difficult task that requires its own study. 

A different situation develops in the case of Hy-coPAI II and Hy-coPAI IV. In each of these cases, 40 mol% more than one of the monomers (diamines) is introduced into the reaction zone. Such features of the reaction should, in any case, lead to the production of copolymers enriched in one of the components since, as shown in our previous article [13], in all cases, the composition of the copolymers corresponds to the amount of components introduced into the reaction. We observed a similar effect in the synthesis of polyamide-imides containing carboxyl groups in the diamine component [11]. Thus, in the case of Hy-coPAI II, the formation of blocks enriched in hydroxyl-containing fragments is possible, and in the case of Hy-coPAI IV, the formation of blocks enriched in fragments that do not contain hydroxyl groups is possible. Apparently, the named areas can be interspersed with statistical fragments.

Studying the synthesized polymers in solutions, including solvents, proved useful as, in this way, polymer film membranes were formed.

According to the static light scattering data, the molecular mass of copolymers *M*_w_ = 38.0–56.7 kDa. The *M*_w_, *A*_2_, *dn*/*dc*, *R*_h-D_, and [*Ƞ*] values obtained for the polymers investigated are given in Table 1.

An analysis of the obtained results showed that a change in the composition of the Hy-coPAI’s is accompanied by a change in their behavior and molecular weight and hydrodynamic characteristics in various solvents.

In dilute solutions of N-MP and in mixed solvents, the behavior and MM characteristics of the copolymers differ. In solutions of copolymers Hy-coPAI’s III (m = 0.5) and Hy-coPAI’s II (m = 0.7), in addition to individual macromolecules, there are aggregates, that is, in N-MP solutions, there were two types of particles: macromolecules with a radius of R_h-f_ and aggregates formed due to the interaction of fragments of chains of different macromolecules of the copolymer with each other. The hydrodynamic radii of *R*_h-s_ aggregates exceeded the sizes of the macromolecules by more than 3–7 orders of magnitude. The aggregates contributed more to total light scattering: the area S_f_ under the peak in the *I* (*R*_h_) distribution curve corresponding to the fast mode was 5–10 times larger than the area S_s_ under the slow mode peak (see Figure 2). However, taking into account the huge difference in the *R*_h-f_ and *R*_h-s_ values, it can be concluded that isolated macromolecules of copolymers prevail in the solution, and our assessment of the proportion of aggregates within the framework of the coil model showed that the concentration of aggregates was no more than 0.5 wt.%, enabling the determination of Mw in this solvent; N-MP itself and mixed solvents are good solvents for this system in terms of thermodynamic quality (see Table 1).

In solutions of homopolymers and copolymer Hy-coPAI IV (m = 0.3), associates cannot be observed via dynamic light scattering (DLS). An increase in the size of associates in the solutions of copolymers from the composition at m = 0.5 (50 mol% fragments of the hydroxyl containing diamine) to the composition at m = 0.7 (70 mol% fragments of the hydroxyl containing diamine) leads to a decrease in the distance between the macromolecular chains in the polymer films, defined in terms of interplanar distances (see below). 

**Figure 5:**
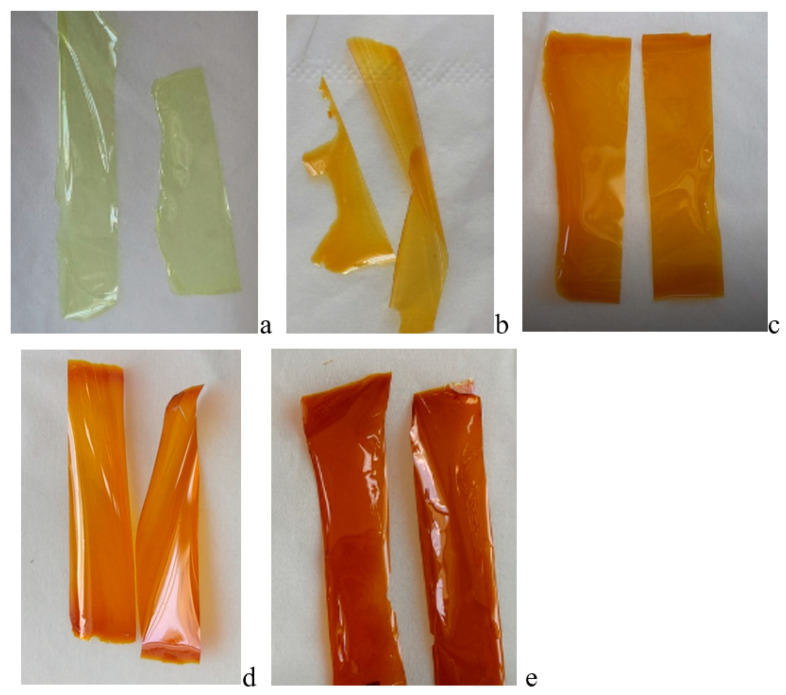
Dense PAI’s (I–V) polymer films: (**a**) PAI V, (**b**) Hy-coPAI IV, (**c**) Hy-coPAI III, (**d**) Hy-coPAI II, (**e**) PAI I.

The synthesized polymers were used to form the non-porous polymer films shown in Figure 5.

It is well-known that the structure of polymer films (or membranes) is formed in the solutions from which they were obtained. This also applies to the packing density of polymer chains. The heterogeneity of macromolecules significantly affects the density of polymers. The nature of the distribution of links along the chain within one macromolecule and the compositional inhomogeneity of the polymer within different macromolecules affect solubility, density, and other properties. The degree of this influence depends both on the nature of the links and on their number. By adjusting the composition of the copolymer, it is possible to obtain polymers in a wide range of densities. 

Table 2 shows that Hy-coPAI III (m = 0.5) is the densest in the series of polymers under consideration, differing by an equal content of units built on the basis of each of the diamines, whereas the homopolymers are the least densely packed. However, the density of PAI V still slightly exceeds the density of PAI I, which is not typical when comparing the homopolymers with and without reactive functional groups. Interestingly, copolymers containing 40 mol% of excess units of one of the two types under consideration have approximately the same density in dilute solutions. However, a copolymer containing 70 mol% of hydroxyl-containing repeating fragments has a higher density in solution than Hy-coPAI IV (m = 0.3). 

These results correlate with the data derived from measuring the density of polymer films (obtained after post-treatment at 150 °C) using the flotation method because the most dense in this series is the copolymer containing 50 mol% of hydroxyl-containing fragments, as shown in Table 2. The densities of Hy-coPAI II (m = 0.7) and Hy-coPAI IV (m = 0.3), in contrast to the solutions, differ significantly in films, and the densest is the copolymer in which hydroxyl-containing fragments predominate. In general, with the exception of Hy-coPAI III (m = 0.5), for all other polymers in this series, the following trend was observed: the density increases with an increase in the content of hydroxyl groups.

It is clear that these properties are determined by the structural features of polymers and the polymer films based on them. 

In the IR spectra of all polymers, the amide group’s imide absorption band characteristics at 1780 cm^−1^ and 1720 cm^−1^, as well as bands at 3290 cm^−1^, 1647 cm^−1^, and 1530 cm^−1^, are notable. At 2920 cm^−1^ and 2850 cm^−1^, there are also bands corresponding to CH_2_ groups in the diamine component. The hydrogen bond band in the spectra of PAI I and the copolymers expands from 3400 cm^−1^ up to 2700 cm^−1^ compared to the PAI V spectrum. This indicates the formation of sufficiently strong hydrogen bonds, probably due to OH groups in the diamine fragment. Moreover, it should be noted that the intensity of the hydrogen bond increases as the percentage of fragments of the hydroxyl-containing diamine increases. Exactly which hydrogen bonds are realized in polymers in the condensed state remains unclear. According to the formula shown in Figure 1, it is possible to form hydrogen bonds between the hydroxyl groups of one macromolecule and the carbonyl groups of another (intermolecular hydrogen bonds), as well as between the hydroxyl groups and carbonyl groups of the amide fragment located in the neighborhood (intramolecular hydrogen bonds). These hydrogen bonds are difficult to distinguish via IR spectroscopy.

**Figure 6:**
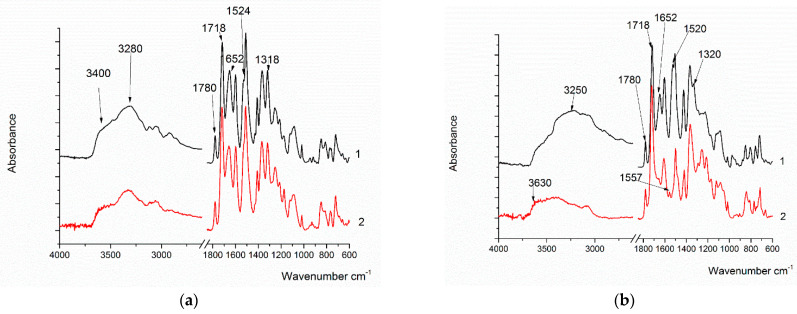

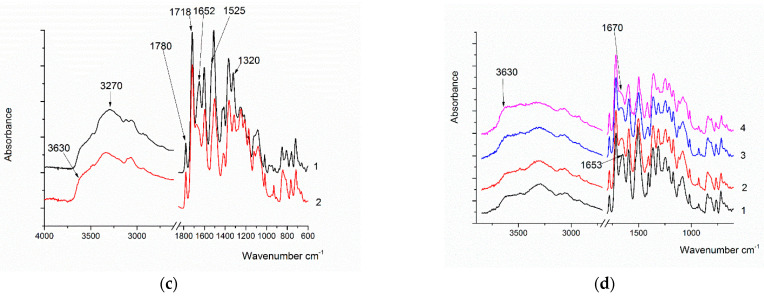
IR spectra of (**a**) PAI V (a1—post-treatment temperature 150 °C, a2—post-treatment temperature 300 °C); (**b**) PAI I (b1—150 °C, b2—300 °C); (**c**) Hy-coPAI III (m = 0.5) (c1—150 °C, c2—300 °C); (**d**) d1—PAI V, d2—Hy-coPAI IV (m = 0.3), d3—Hy-coPAI III (m = 0.5), d4—Hy-coPAI II (m = 0.7); d1, d2, d3, d4—post-treatment temperature 300 °C.

The synthesized (co)polymers in the form of powders were also studied via IR spectroscopy in the ATR mode (Figure 6). In the PAI-V sample (Figure 6a), absorption bands characteristic of PAI were observed with frequencies in the regions of 1780 cm^−1^, 1718 cm^−1^ (related to the symmetric and antisymmetric vibrations of the C = O group of the imide ring), a band in the region of 1370 cm^−1^ (stretching vibrations C-N), 725 cm^−1^ (C = O bending vibrations in the cycle), and absorption bands at 1644 cm^−1^ (C = O absorption) and 1524 cm^−1^ (NH bending vibrations) in relation to the amide group. The broad band with a maximum frequency at 3400 cm^−1^ includes the stretching vibrations of the bound NH group of the amide part of the macromolecule and the OH groups.

After sample heating, a slight decrease in the intensity of the band at 3400 cm^−1^ and the band of Amide I at 1652 cm^−1^ was observed in the spectrum, which is probably due to the removal of solvent residues. There were no significant changes.

In the PAI I sample, absorption bands characteristic of PAI, which are related to the imide and amide parts of the copolymer, were also observed. However, it should be noted that the band at 3400 cm^−1^ shifts to 3200 cm^−1^, which is typical for the formation of the stronger hydrogen bonds of the OH groups. It is probable that the hydroxyl groups associated with the aromatic ring of the amide part of the molecule are involved in the formation of these bonds. 

**Figure 7:**
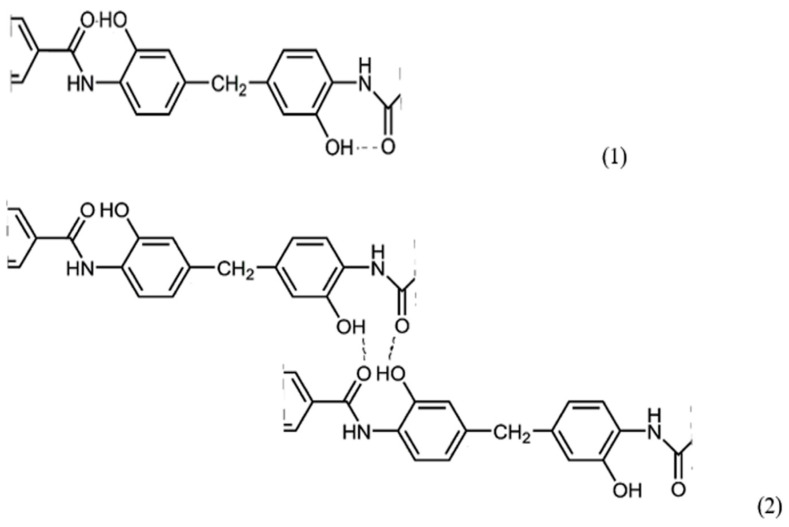
Two possible ways of hydrogen bonding in (co)polymers containing fragments of diamine DADHyDPhM.

The chemical structure of the synthesized polymers, except for PAI-V, suggests the possibility of the formation of intra- (1) and intermolecular (2) hydrogen bonds, as shown in Figure 7. 

**Figure 8:**
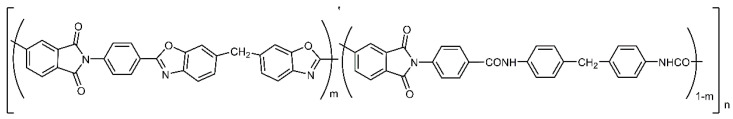
Chemical structure of (co)polymers annealed at 300 °C.

From the sketch in Figure 7, it can be observed that, in these polymer systems, the implementation of an intramolecular hydrogen bond is most likely, although the occurrence of an intermolecular one cannot be ruled out either. Although it is difficult to determine which particular hydrogen bond is present in the sample via IR spectroscopy, it made sense to use this method to confirm hydrogen bonds in polymers with different contents of hydroxyl groups. This is all the more important since the intramolecular hydrogen bond is a precursor of the chemical bond formed during the formation of the cycle as a result of the thermal dehydrocyclization reaction with the formation of the compounds of the following formula (Figure 8):

During heating, the main amide bands—Amide I (1652 cm^−1^), Amide II (1525 cm^−1^), and Amide III (1320 cm^−1^)—almost completely disappear and, corresponding to the vibration of the C=N group located in a cycle or conjugate, a band at 1557 cm^−1^ appears. These changes confirm the formation of the putative ring structure instead of the amide bond. The band at 3200 cm^−1^ almost completely disappears, and the band at 3630 cm^−1^, which is characteristic of free hydroxyl groups, begins to appear. Thus, it can be assumed that the water released during cyclization is retained in the molecules, but at the same time, it remains in an isolated state.

In sample Hy-coPAI III (m = 0.5), upon heating, a decrease in the intensities of the Amide I (1652 cm^−1^), Amide II (1525 cm^−1^), and Amide III (1320 cm^−1^) bands was observed. The band at 1557 cm^−1^ is faintly visible against the background of the remaining Amide II band. In addition, the Amide I band (1652 cm^−1^) shifts to 1660 cm^−1^ upon heating. In all likelihood, this is due to the fact that intermolecular interactions significantly contribute to the position of the absorption band of Amide I, and when a significant part of the amide groups disappears from the polymer molecule, these interactions decrease significantly.

The IR spectra of the films of the PAI’s copolymers with different component ratios that had been heated to 300 °C were also recorded and compared. As the OH group’s content in the sample increases, the intensity of the band at 1557 cm^−1^ increases, and the shift of the Amide I band from 1653 cm^−1^ to 1670 cm^−1^ becomes stronger, which also indicates a weakening of intermolecular interactions.

The thermal treatment should lead to the removal of adsorbed water from the polymer film and promote the possible formation of hydrogen bonds, the presence of which has been clearly demonstrated by our IR spectral analysis of dense films discussed above.

In order to establish the thermophysical characteristics of coPAI containing hydroxyl groups and the initial PAI homopolymers, TGA, DSC, and DMA were used to study the samples that had been heat-treated at 150 °C (it was for such membrane samples that a series of pervaporation tests were carried out) and at 300 °C, for which there was partial hydrocyclization. The structural formulas of coPAI are shown in Figure 1.

**Figure 9:**
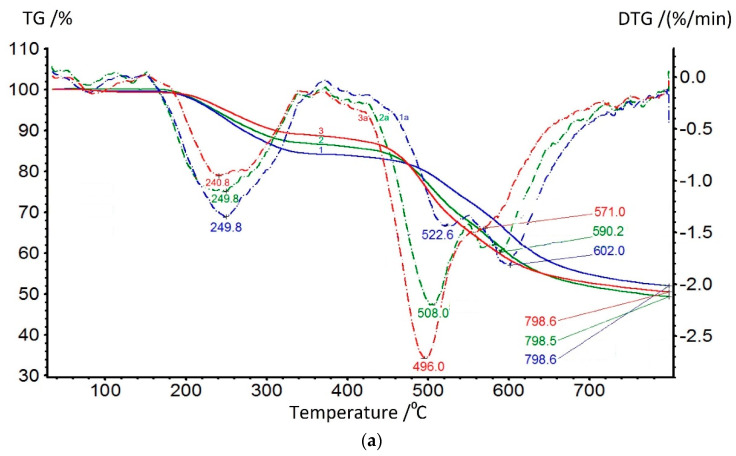

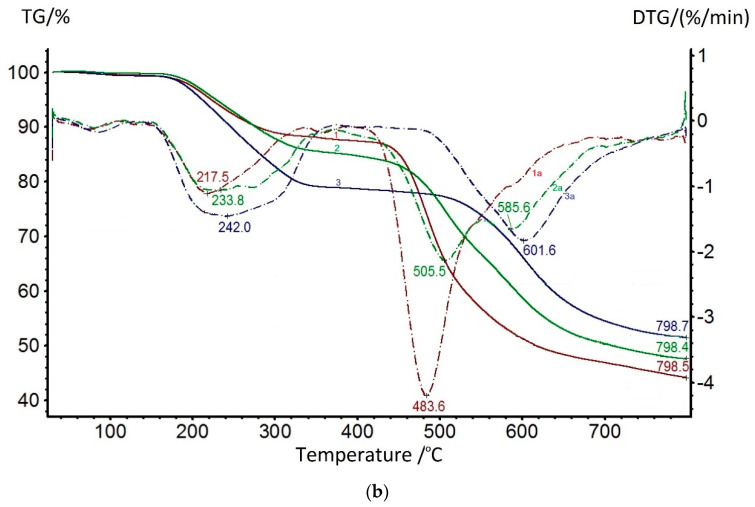
(**a**) TGA (solid line:1—Hy-coPAI II, 2—Hy-coPAI III, 3—Hy-coPAI IV) and DTG curves (dotted line: 1a—Hy-coPAI II, 2a—Hy-coPAI III, 3a—Hy-coPAI IV) of Hy-coPAIs samples; (**b**) TGA (solid line: 1—DADPhM, 2—Hy-coPAI III (m = 0.5), 3—DADHyDPhM) and DTG curves (dotted line: 1a—DADPhM, 2a—Hy-coPAI III (m = 0.5), 3a—DADHyDPhM) of homopolymer samples.

In Figure 9a, the TGA data of coPAI samples with different contents of the hydroxyl-containing fragment are presented. At up to 150 °C, adsorbed water is released; its comparative amount can be determined from DSC data. Further, in the range of 150–320 °C, a residual solvent is released, the amount of which, according to TGA data, increases as the component containing hydroxyls in the copolymer increases. The temperature of the maximum rate of this process varies slightly depending on the coPAI composition, but its slight increase from 240 to 249 °C as the hydroxyl-containing fragment in co-PAI increases. The process of releasing the residual solvent is accompanied by the process of final cyclization with coPAI, which is more reflected in the DTG curves: the release of water during the formation of a ring in the cyclization of a part of a copolymer containing hydroxyl (-OH groups in the diamine component in the o-position to the amide bond) is superimposed on the end of the residual solvent extraction process, which is very clearly seen in the DTG curves shown in Figure 9a.

The onset of the thermal decomposition of the main chain is recorded above 420 °C and is bimodal for all three coPAIs; the maxima of these processes shift towards higher temperatures as the content of the DADHyDPhM component increases, and this is consistent with previous studies on the thermal stability of powder compositions [39]. The bimodality of the coPAI thermal decomposition data is associated with the sequential thermal decomposition of its constituent homopolymers, which is confirmed by the TGA data shown in Figure 9b (the maximum decomposition rates for both DADPhM and DADHyDPhM homopolymers are recorded at 483 and 601 °C, respectively). Consequently, the block with the hydroxyl-containing fragment (DADHyDPhM) decomposes last in coPAI at higher temperatures. Attention should be paid to the extraction of the residual solvent from homopolymers. For the PAI V homopolymer, if this process is a one-stage process with the temperature of the maximum solvent release rate being 217 °C, then the process of solvent extraction from the PAI I homopolymer is broadened (due to the hydrocyclization process being superimposed on it). In this case, the temperature of the maximum solvent release rate increases to 242 °C. The described processes are divided for the Hy-coPAI III (m = 0.5) (Figure 9b).

**Figure 10:**
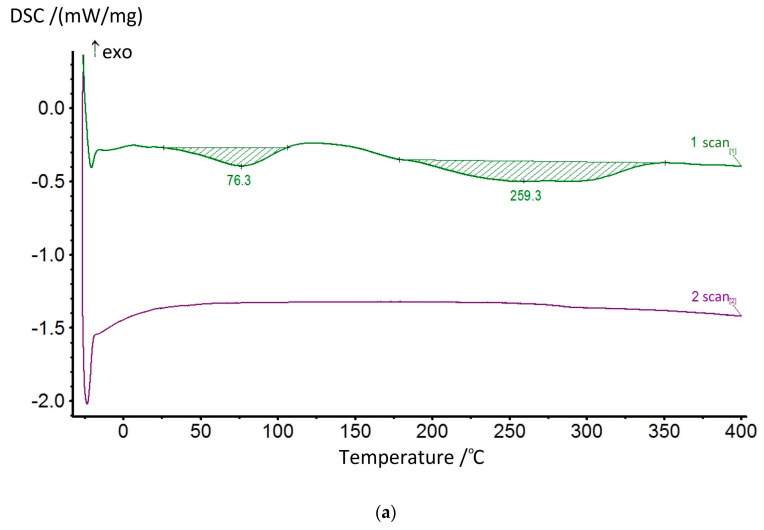

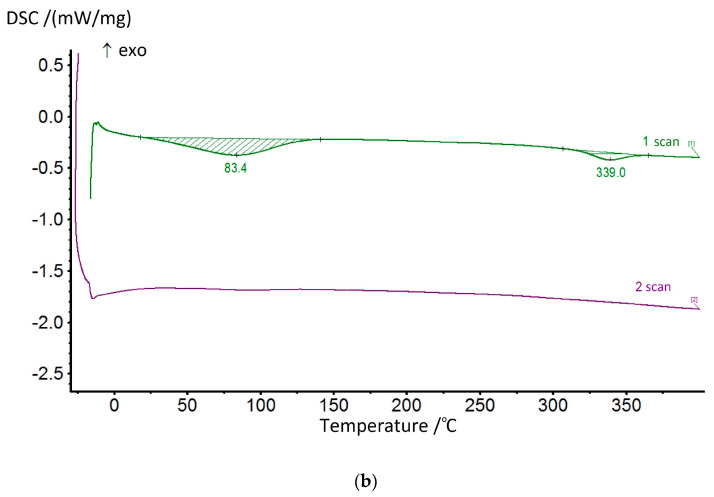
DSC curves of Hy-coPAI III (m = 0.5) samples heat-treated at 150 °C (**a**) and heat-treated at 300 °C (**b**).

The dehydrocyclization process was studied in more detail by using the DSC method. Since this process involves significant energy consumption, the studies were carried out for the samples of compositions and homopolymers of non-cyclized fragments in ascending order. The most typical thermograms for the coPAI samples processed at different temperatures are shown in Figure 10. During the first scan, two endotherms were observed on the DSC curves, reflecting the release of adsorbed water and, at higher temperatures, the completion of the cyclization process of fragments containing hydroxyl groups. The high-temperature endotherm may represent a superposition of two processes: residual solvent release and cyclization (Figure 10a). In this case, the endotherm has a bimodal character and is stretched in temperature (the evaporation temperature of N-Methyl-2-pyrrolidone is 202 °C). After heating the coPAI above 200 °C for an hour, the release of the residual solvent was not recorded.

The value of the enthalpy of the residual dehydrocyclization process decreases with an increase in the sample pretreatment temperature and a decrease in the amount of hydroxyl-containing fragments with coPAI. The results of the studies are shown in Table 3.

Thus, at treatment temperatures above 200 °C, the value of the enthalpy of the second endotherm, where the dehydrocyclization process prevails, changes symbatically with the content of the hydroxyl-containing fragment and is 20.35, 52.65, and 139.6 J/g for compositions Hy-coPAI IV, Hy-coPAI III, Hy-coPAI II, respectively. In the presence of the residual solvent (for example, at a final processing temperature of 150 °C), these values increase and are 62.27, 90.25 and 156.5, respectively. Noteworthy is the value of the enthalpy of the endotherm of the composition 50:50 after heat treatment at 300 °C. This value is the maximum for all three compositions, which corresponds to the maximum value of the number of non-cyclized units for the compositions. Another anomaly of this composition is a significant increase in free water as the processing temperature of the composition increases, which may be due to a change in free volume. The enthalpies of water release for the Hy-coPAI IV and Hy-coPAI II compositions are approximately the same and close to those for the DADHyDPhM homopolymer (treated at the same temperature of 300 °C) and significantly lower than for the Hy-coPAI III composition (see Table 3). A great amount of attention has been paid to the data pertaining to the samples processed at high temperatures due to the need to analyze the results of our research using dielectric spectroscopy.

**Figure 11:**
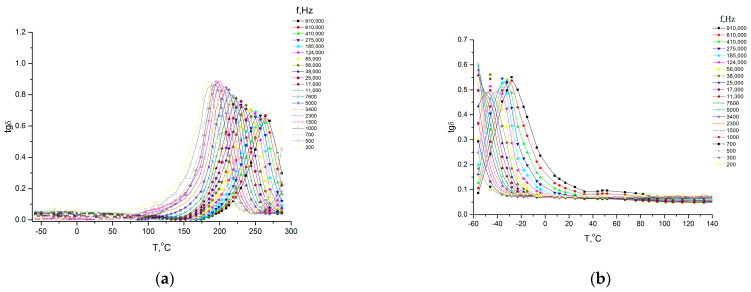
Temperature–frequency dependences of the dielectric loss tangent (tgδ) for homopolymers based on DADPhM ((**a**) PAI V) and DADHyDPhM ((**b**) PAI I).

**Figure 12:**
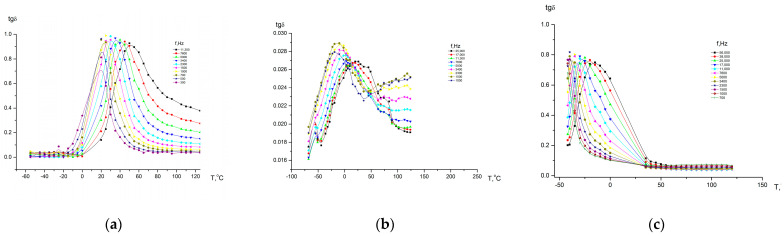
Temperature–frequency dependences of the dielectric loss tangent (tgδ) for copolymers based on DADHyDPhM and DADPhM. (**a**) Hy-coPAI IV (m = 0.3); (**b**) Hy-coPAI III (m = 0.5); (**c**) Hy-coPAI II (m = 0.7).

All polymers studied via dielectrospectroscopy (Figure 11 and Figure 12) were heated to 300 °C in order to complete the cyclization process as fully as possible. However, as the IR spectroscopy data show (see above), a small amount of hydroxyl groups remained.

**Figure 13:**
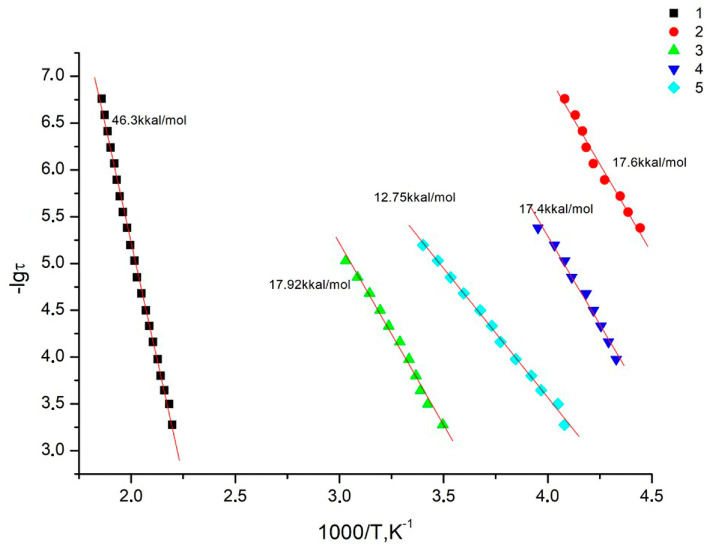
Dependences of relaxation time on reciprocal temperature for all studied systems: 1—PAI V; 2—PAI I; 3—Hy-coPAI IV (m = 0.3); 4—Hy-coPAI III (m = 0.5); 5—Hy-coPAI II (m = 0.7).

In the studied temperature–frequency range, all systems exhibit one relaxation process. As can be seen from Figure 13, the dependences *lgτ_ℳaκc_* = φ(1/*T*) are linear, which is typical for local forms of mobility, i.e., they are characterized by an environment-independent jump of polar kinetic units from one energy equilibrium state to another. In this case, the dependences *lgτ_ℳaκc_* = φ(1/*T*) are linear and obey the Arrhenius equation (assuming a unique relaxation time):

*τ_ℳaκc_*(*T*)= *τ_o_*exp(*E_a_* /k*T*), (3)

where *E_a_* is the activation energy of the process, k is the Boltzmann constant, and *τ_o_* is the pre-exponential and reflects the relaxation time at an infinitely high temperature.

The relaxation processes in the systems under consideration are most likely associated with fluctuations in the kinetic units in the main chain of homopolymers or random copolymers (the process is realized at different temperatures depending on the composition of the copolymer), but according to the reaction conditions, a block copolymer should be obtained.

**Figure 14:**
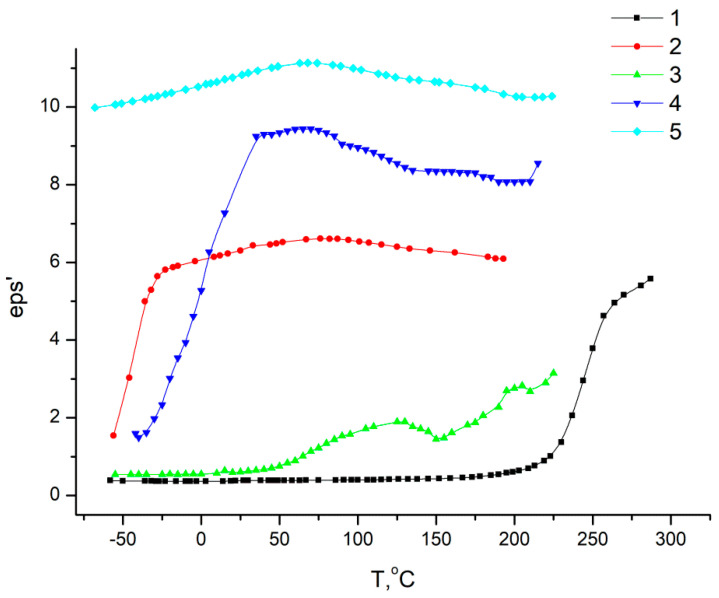
Dependence of dielectric constant on temperature for all studied systems (f = 38 kHz). 1—PAI V; 2—PAI I; 3—Hy-coPAI IV (m = 0.3); 4—Hy-coPAI III (m = 0.5); 5—Hy-coPAI II (m = 0.7).

According to the data shown in Figure 14, for homopolymers, relaxation processes are observed characteristics of these polymers, and for copolymers, in a 0–200 °C temperature range, dependences are observed, the course of which differs significantly from that which is characteristic of this class of polymers. This is due to the fact that homopolymers differ in their dielectric constant (eps’). At the boundaries of regions in which fragments of macromolecules of one or another homopolymer predominate, McChwell/Wagner polarization may occur [39,40]. In this regard, since we observed a combination of effects, it is difficult to explain the dependences of the dielectric constant on temperature from the point of view of considering relaxation transitions. 

The data presented above directly and indirectly affirm the block structure of copolymer macromolecules and the presence of two types of hydrogen bond. This leads to the absence of the direct dependence of hydrophilicity on the number of hydroxyl groups. As we have shown in [13], the presence of the residual solvent can affect the properties of (co)PAI films. The assumption that the pervaporation properties of membranes obtained at 100 °C can be determined by the presence of a residual solvent in the polymer film was confirmed in the present study. However, as shown in this work, this is not the only factor that determines the transport properties of membranes. 

The data obtained as a result of preliminary testing during the pervaporation process indicated that thermally treating the films (annealing), preferably up to 150 °C, is necessary for stable results. Indeed, according to the IR spectra, after annealing up to 150 °C, there was no change in the chemical structure of polymers. From our previous studies, it is known that the N-MP solvent is firmly retained in the matrix of polyamide-imides, forming complexes with polymers of this class [35]. The strength of these complexes results in PAI-based polymer film membranes retaining their properties during long-term storage. In this case, the residual solvent plays the role of a plasticizer and contributes to the good mechanical characteristics of polymer films.

Table 2 and Table 4 present the results of studying films formed by heating to 150 °C under the same conditions. These samples are characterized by the presence of N-MP, the significant release of which begins at 200 °C, as shown by DSC (Figure 10). Membrane samples obtained by post-treating the formed films by annealing at 150 °C were studied during pervaporation. The films were tested by passing individual substances through them at 40 °C in the following order: cyclohexane (I), ethanol (I), methanol (I), water (I), water (II), methanol (II), ethanol (II), cyclohexane (II). The results obtained are presented in Table 4.

The data presented in Table 4 shows that all samples heated to 150 °C have high methanol permeability, which exceeds the permeability of both water, ethanol (polar solvents), and cyclohexane (non-polar solvent). At the same time, PAI I, Hy-coPAI II (m = 0.7), and PAI V (N 1, 3, 12 in Table 4) are the most permeable for methanol. 

Regarding ethanol permeability, it really increases with an increase in the concentration of hydroxyl groups in the polymer (an increase in the series 12, 8, 3, 1 of Table 4). A similar trend is observed when ethanol or water is passed through the membranes, if we assume that the water permeability values of Hy-coPAI II (m = 0.7) and Hy-coPAI III (m = 0.5) are close.

All membranes have a significantly lower cyclohexane permeability. However, in this case, there is no clear dependence on the number of hydroxyl groups in macromolecules. Interestingly, the cyclohexane permeability of the denser PAI I polymer film is ~two times greater than that of PAI V, according to Table 2 and Table 4. This is possible only because of the stronger interaction between cyclohexane and PAI I than PAI V. In the case of cyclohexane, we can only talk about hydrophobic interactions. However, this seems odd since PAI I has two hydroxyl groups for each repeat unit. This effect is possible only when almost all hydroxyl groups take part in the formation of hydrogen bonds, in accordance with Figure 7. In the environment of cyclohexane, where the interaction between it as a poor solvent and a hydroxyl-containing polymer is extremely small, the formation of an intramolecular hydrogen bond is realized, while the macromolecule conformation changes. With a change in the conformation of the polymer in membranes, a change in the free volume is also possible, which contributes to an increase in permeability to cyclohexane. 

Special attention is paid to Hy-coPAI IV (m = 0.3), which, when obtained at a temperature of 100 °C, is stable and effective in pervaporation processes, but it is not stable in the case of a post-treatment temperature of 150 °C. Apparently, this is due to the presence of the residual solvent and the effect of the plasticization of the polymer film. The film heated to 150 °C, from which the largest amount of N-MP was removed, was compacted and embrittled. It remains unclear why this effect is typical only for this copolymer, whose structure is dominated by 40 mol% fragments that do not contain hydroxyl groups.

It should be noted that all copolymers heated at 150 °C were less stable than homopolymers in the transmembrane transfer of cyclohexane or ethanol. At the same time, the block copolymers Hy-coPAI II (m = 0.7) and Hy-coPAI IV (m = 0.3) are not stable during the mass transfer of methanol, and the mostly random copolymer Hy-coPAI III (m = 0.5) is unstable mainly with respect to cyclohexane. The latter effect manifests itself only if the membrane has been previously exposed to polar liquids that have penetrated into the intermolecular space. If only cyclohexane was passed through this membrane, its integrity is not disturbed.

Hy-coPAI II (m = 0.7) is the most selective of the considered polymers obtained at 150 °C. However, with a successive mass transfer of penetrants, as their polarity decreases, the stability of the membrane decreases. The copolymer, as discussed above, has 40 mol% more hydroxyl-containing diamine fragments. In this case, the presence of residual N-MP can facilitate the penetration of polar liquids. This is consistent with the data in Table 1 (presented above).

Table 4 shows a comparison of the data on the permeability of the Hy-coPAI II samples (m = 0.7) obtained upon annealing up to temperatures of 150 °C, 200 °C, and 300 °C. The transition to the sample Hy-coPAI II (m = 0.7; 200 °C) is accompanied by the removal of residual N-MP, as shown in Table 3 and Figure 9 and Figure 10. This leads to a significant decrease in permeability to polar liquids and an increase in permeability to cyclohexane (N5 Table 4). Further increases in post-treatment temperature results in a cyclization reaction to form the polymers shown in Figure 8, which is detected for all polymers by the IR spectra of Figure 6d. The permeability of the resulting membrane for all penetrants (N6 Table 4) is significantly lower than all membranes formed at lower temperatures. Apparently, this is due to a change in the structure of the membrane. Although this effect has been described using Hy-coPAI II (m = 0.7; 300 °C) as an example, it applies to all of the hydroxyl group-containing membranes in this series. The structural features of (co)PAI (300 °C) appear in our IR spectral analysis (Figure 6e), dielectric spectroscopy (Figure 13 and Figure 14), and X-ray analysis data (Table 5).

Hydroxyl-containing copolyamide-imides (DADHyDPhM:DADPhM) before and after the pervaporation process were studied by wide-angle X-ray diffraction (WAXRD). The results are presented in Table 5. As can be seen for the membranes based on DADPhM being heat-treated at 100 °C, there is an insignificant shift in the amorphous halo maximum toward larger angles, which corresponds to a decrease in the interplanar spacing (from 4.84 to 4.68 Å).

At the same time, a reflex appears at angle 2θ = 15.55°, with interplanar spacing equal being to 5.69 Å. The trend towards a decrease in interplanar distances after the pervaporation process remains for this PAI for the heat-treated samples and at 150 °C. This pattern is also characteristic of the DADHyDPhM homopolymer, albeit in a less pronounced manner.

The results of processing the diffraction patterns of the coPAI’s membranes at various ratios of DADHyDPhM and DADPhM are also given in Table 5. It is interesting to note that, as the hydroxyl-containing fragments increase in the DADHyDPhM:DADPhM series (DADHyDPhM 30, 70, and 100%), a decrease in the interplanar distance d, Å, corresponding to the maximum of the amorphous halo, which correlates with the distance between the macromolecular chains of homo and coPAI’s, can be observed. This dependence becomes especially noticeable after the heat treatment of the samples at 150 °C. The decrease in interplanar spacing can be explained by possible hydroxyl crosslinking between macromolecular chains. Hy-coPAI III data (50:50) fall out of such dependence. The interplanar spacing value for this coPAI turned out to be less (see Table 5) than the value corresponding to Hy-coPAI II (m = 0.7), which should lead to an increase in the density of the Hy-coPAI III (m = 0.5) sample. The same anomaly for Hy-coPAI III (m = 0.5) was also observed for the densities obtained by using the flotation method (Table 2). The data obtained also correlate well with an increase in the density of film membranes with an increase in hydroxyl-containing fragments.

The presence of reflections, in addition to the amorphous halo (see Table 5), allows us to conclude that most of the homo and coPAI’s samples are in an ordered mesomorphic or crystalline state. The data are also confirmed by the results of the study involving the method of polarization microscopy (Boethius-type stage); in crossed Nicols, the films glow; the samples are anisotropic.

## 4. Conclusions

For the present study, polyamide-imides differing in the number of hydroxyl groups in diamine fragments of polymer chains were synthesized by low-temperature (co)polycondensation. Due to changes in the conditions for introducing monomers into the reaction zone, polymers with different distributions of repeating fragments in macro-chains were obtained. This determined the behavior of polymers both in solutions and in films formed from these solutions. A change in copolymer composition is accompanied by some changes in their molecular weight and hydrodynamic characteristics in various solvents. In solutions of Hy-coPAI’s III (m = 0.5) and Hy-coPAI’s IV (m = 0.7) copolymers, in addition to individual macromolecules, aggregates were present in small amounts, that is, two types of particles were present in N-MP solutions: macromolecules with a radius R_h-f_ and aggregates formed due to the interaction of chain fragments of different copolymer macromolecules with each other. The hydrodynamic radii of R_h-s_ aggregates exceeded the sizes of macromolecules by more than 3–7 orders of magnitude. An increase in the size of associates in copolymer solutions upon passing from Hy-coPAI’s III (50 mol% hydroxyl-containing fragments) of diamine) to Hy-coPAI’s IV (70 mol% hydroxyl-containing fragments) corresponds to a decrease in the distance between macromolecular chains in polymer films, determined through interplanar distances. The densities of the homopolymers are close, but the density of PAI V still slightly exceeds the density of PAI I, which is atypical when comparing homopolymers with and without reactive functional groups. An analysis of the data obtained suggested that, in these systems, the implementation of an intramolecular hydrogen bond, which promotes the dehydrocyclization of polymers when heated to 300 °C and above, is most advantageous. Both processes lead to a decrease in the hydrophilicity of polymers and conformational rearrangements. Following an investigation into the structural analysis data obtained via X-ray diffraction analysis, IR spectroscopy, and dielectric spectroscopy, as well as thermal analysis, how polymer films are formed in the presence of a residual solvent was shown, as was its subsequent removal through various methods (thermally or via successive pervaporation). The results obtained are not only of general scientific importance but are also important in practice in the formation of membranes with predictable properties. The resulting membrane samples showed high methanol permeability along with high selectivity with respect to nonpolar liquids. This effect is associated with the presence of a strongly retained N-MP in the polymer film.

## Figures and Tables

**Table 1:** Molar mass and hydrodynamic characteristics of polymers.

*Sample*	*Solvent*	*dn*/*dc*, cm^3^·g^−1^	M_w_, kDa	*A*_2_, cm^3^⋅mol⋅g^−2^	R_h-f_, nm	R_h-s_, nm	[*Ƞ*], cm^3^·g^−1^
PAI V	N-MP	0.2141	42.6	1.39 × 10^−3^	5.4	-	86.0
PAI I	N-MP	0.1977	41.7	3.16 × 10^−3^	5.6	-	85.7
	N-MP/*EtAc = 70/30 (vol%)	0.2794	44.9	2.30 × 10^−3^	5.5	-	87.3
	N-MP/*MeOH = 94/6 (vol%)	0.2061	49.6	3.19 × 10^−3^	4.8	-	78.0
Hy-coPAI IV (m = 0.3)	N-MP	0.1923	38.0	1.72 × 10^−3^	4.8	-	53.0
Hy-coPAI III (m = 0.5)	N-MP	0.2143	48.7	9.02 × 10^−3^	5.6	114	54.9
Hy-coPAI II (m = 0.7)	N-MP	0.2216	56.7	4.81 × 10^−3^	6.5	446	56.6

*EtAc—Ethyl Acetate. *MeOH—Methanol.

**Table 2:** Density measurement results. Specific partial volume of (co)polymers in N-MP. Polymer film density ρ*.

N	Sample	1-v_2·_p	v_2_, cm^3^ /g Specific Partial Volume	ρ*, g/cm^3^
1	PAI-1	0.1122	0.8605	1.3573
2	Hy-coPAI II (m = 0.7)	0.2495	0.7274	1.3429
3	Hy-coPAI III (m = 0.5)	0.4205	0.5617	1.3577
4	Hy-coPAI IV (m = 0.3)	0.2092	0.7665	1.3323
5	PAI-V	0.1554	0.8186	1.3173

ρ_0_ = 1.0317 (N-MP)—solvent density, g/cm^3.^

**Table 3:** Results of our studies on the compositions and homopolymers using DSC *.

Sample	Processing Temperature, °C	T_max_, °C Endotherm 1	ΔH, J/g Endotherm 1	T_max_, °C Endotherm 2	ΔH, J/g Endotherm 2
PAI I (m = 1)	300	73.0	40.35	336	20.68
Hy-coPAI II (m = 0.7)	300	78.7	40.84	340.2	4.54
	250	81.2	39.78	297.8	139.6
	200	87.3	37.08	293.2	130.8
	150	78.2	58.75	228; 302	156.5
Hy-coPAI III (m = 0.5)	300	83.0	60.47	339	10.94
	250	84.0	42.31	313	52.65
	200	89.0	34.19	292	70,75
	150	76.0	30.93	259	90.35
Hy-coPAI IV (m = 0.3)	300	111.7	34.03	343.0	6.24
	250	100.0	31.56	311.7	20.35
	200	86.7	34.16	287.1	61.31
	150	88.5	30.63	286.4	62.27
PAI V (m = 0)	300	84.3	60.51	-	-

* The glass transition temperatures of the initial homopolymers and copolymers were not recorded using DSC.

**Table 4:** Pervaporation properties (P [µm⋅kg⋅m^−2^⋅h^−1^]) of dense membranes at 40 °C. The order of penetrants during pervaporation: (I) Cyh → EtOH → MeOH → HOH; (II) HOH → MeOH → EtOH → Cyh.

N	Membrane	* T, °C	P, kg·µm·m^−2^h^−1^				α *** (Methanol/Cyh)	Penetrants Order
			** Cyh	Ethanol	Methanol	Water		
1	PAI I	150	0.074 ± 0.004	1.34 ± 0.07	2.31 ± 0.11	0.504 ± 0.025	31	→ (I)
2			0.063 ± 0.003	0.100 ± 0.005	0.840 ± 0.042	0.767 ± 0.038	13	← (II)
3	Hy-coPAI II (m = 0.7)	150	0.018 ± 0.001	0.216 ± 0.011	2.20 ± 0.11	0.205 ± 0.010	122	→ (I)
4			-	swollen, damaged after drying	0.933 ± 0.047	0.162 ± 0.008	-	← (ΙΙ)
5		200	0.285 ± 0.014	0.024 ± 0.001	0.033 ± 0.002	0.165 ± 0.008	0.12	→ (I)
6		300	0.015 ± 0.001	0.012 ± 0.001	0.015 ± 0.001	0.042 ± 0.002	1	→ (I)
7						0.057 ± 0.003	-	← (ΙΙ)
8	Hy-coPAI III (m = 0.5)	150	0.044 ± 0.002	0.082 ± 0.004	0.843 ± 0.042	0.292 ± 0.015	19	→ (I)
9			swollen, damaged after drying	0.126 ± 0.006	0.663 ± 0.033	0.401 ± 0.020	-	← (ΙΙ)
10	Hy-coPAI IV (m = 0.3)	100	0.122 ± 0.006	0.450 ± 0.023	3.08 ± 0.154	0.269 ± 0.013	25	→ (I)
			0.180 ± 0.009	0.302 ± 0.015	2.18 ± 0.11	0.596 ± 0.030	12	← (ΙΙ)
11		150	0.0040 ± 0.0002	swollen, damaged after drying	-	-	-	→ (I)
12	PAI V		0.034 ± 0.002	0.013 ± 0.001	1.54 ± 0.08	0.056 ± 0.003	45	→ (I)
13		150	0.014 ± 0.001	0.063 ± 0.003	0.999 ± 0.050	0.176 ± 0.008	71	← (ΙΙ)

* T—Post-treatment temperature; ** Cyh = Cyclohexane; α ***—ideal permselectivity.

**Table 5:** Scattering angles (2θ) and interplanar distances (d) of homopolymers and coPAI’s depending on the treatment temperature and the amount of hydroxyl-containing fragments.

N Sample	DADHyDPhM:DADPhM	* T, °C	2θ, Degree		*d*, Å	
				*halo*		*halo*
PAI V before PV	0:100	100	8.05	18.3	10.97	4.84
PAI V after PV			15.55	18.9	5.69	4.68
PAI V before PV		150		18.4		4.81
PAI V after PV			16.05	19.4	5.52	4.57
PAI I before PV	100:0	100		21.8		4.07
PAI I after PV			16.81	21.8	5.27	4.07
PAI I before PV		150	17.34	23.0	5.11	3.86
PAI I after PV			16.71	23.3	5.30	3.81
Hy-coPAI IV before PV	30:70	100		17.9		4.96
Hy-coPAI IV after PV				17.9		4.94
Hy-coPAI IV before PV		150	4.14	17.8	21.31	4.97
Hy-coPAI IV after PV				20.2		4.39
Hy-coPAI III before PV	50:50	100		17.9		4.95
Hy-coPAI III after PV				17.9		4.94
Hy-coPAI III before PV		150	16.86 19.71 21.48	18.7	5.25 4.50 4.13	4.74
Hy-coPAI III after PV			-	-	-	-
Hy-coPAI II before PV	70:30	100		19.3		4.60
Hy-coPAI II after PV			14.06	18.2	6.08	4.82
Hy-coPAI II before PV		150	4.08	18.2	21.62	4.84
Hy-coPAI II after PV			-	-	-	-

* T—Post-treatment temperature.

## Data Availability

Data is contained within the article.
